# Interaction of cholinergic disruption and age on cognitive flexibility in rats

**DOI:** 10.1007/s00221-022-06472-x

**Published:** 2022-10-05

**Authors:** Celine Cammarata, Eve D. De Rosa

**Affiliations:** 1 Department of Psychology, Cornell University, Ithaca, NY 14853 USA; 2 Human Neuroscience Institute, Cornell University, Ithaca, NY 14853 USA; 3 Department of Neurobiology, Duke University School of Medicine, Durham, NC 27710 USA

**Keywords:** Acetylcholine, Cognitive aging, Cognitive flexibility, Executive function

## Abstract

Healthy aging is associated with a functional reduction of the basal forebrain (BF) system that supplies the neurochemical acetylcholine (ACh) to the cortex, and concomitant challenges to cognition. It remains unclear how aging and ACh loss interact to shape cognition in the aging brain. We used a proactive interference (PI) odor discrimination task, shown to depend on the BF in young adults, wherein rats acquired new associations that conflicted with past learning or associations that did not conflict. This manipulation allowed independent assessment of encoding alone vs. encoding in the face of interference. Adult (9.8 ± 1.3 months) or aged male Long-Evans rats (20.7 ± 0.5 months) completed the PI task with systemic administration of a muscarinic cholinergic antagonist, scopolamine, or a pharmacological control. Aged rats were less able to resolve PI than adult rats. Moreover, while scopolamine reduced efficient PI resolution in adult rats, this cholinergic antagonism had no additional effect on aged rat performance, counter to our expectation that scopolamine would further increase perseveration in the aged group. Scopolamine did not impair encoding of non-interfering associations regardless of age. These data suggest that natural aging changes the effect of cholinergic pharmacology on encoding efficiency when past learning interferes.

## Introduction

Advancing age typically brings challenges to cognitive flexibility, the capacity to adaptively encode new information in a changing environment (Barense et al. 2002; Harada et al. 2013; Head et al. 2009; Lacreuse et al. 2018). This is captured in the phenomenon of proactive interference (PI), the impairment of acquiring new associations that overlap or conflict with past learning: whereas younger adults are typically able to resolve PI readily, aged rodents (Winocur 1984) and humans (Pettigrew and Martin 2014) are slower and less successful in overcoming interference. Concurrently, the basal forebrain (BF) cholinergic system, which supplies the modulatory neurochemical Acetylcholine (ACh) throughout the cerebral cortex, demonstrates reduced function over the lifespan, contributing to natural cognitive aging (Fischer et al. 1992; Gibson et al. 1981; Lammers et al. 2018; Luine et al. 1990; Wolf et al. 2014).

The BF cholinergic system is critical to key aspects of cognitive flexibility such as learning new stimulus–response associations (Chen et al. 2004), the dynamic allocation of attention (Botly and De Rosa 2007, 2008, 2009, 2012; Bucci et al. 1998; Ljubojevic et al. 2014, 2018) and juggling multiple stimulus–response associations (McGaughy et al. 1994). One model suggests that ACh neuromodulation promotes encoding of new information over retrieval or consolidation of prior learning by simultaneously enhancing sensory input to the cortex and hippocampus while suppressing recurrent activity within the cortical circuitry (Hasselmo and McGaughy 2004). Moreover, while reducing ACh or bolstering ACh activity can impair or enhance flexible re-learning of rules and associations, respectively, such manipulations do not impact the ability to initially learn associations (Chen et al. 2004; Nikiforuk et al. 2015; Seeger et al. 2004; Wood et al. 2016). This suggests that ACh is not essential for all learning but rather is specifically recruited when subjects must update previously acquired information and resolve the resulting conflict between past and present associations.

**Fig. 1:**
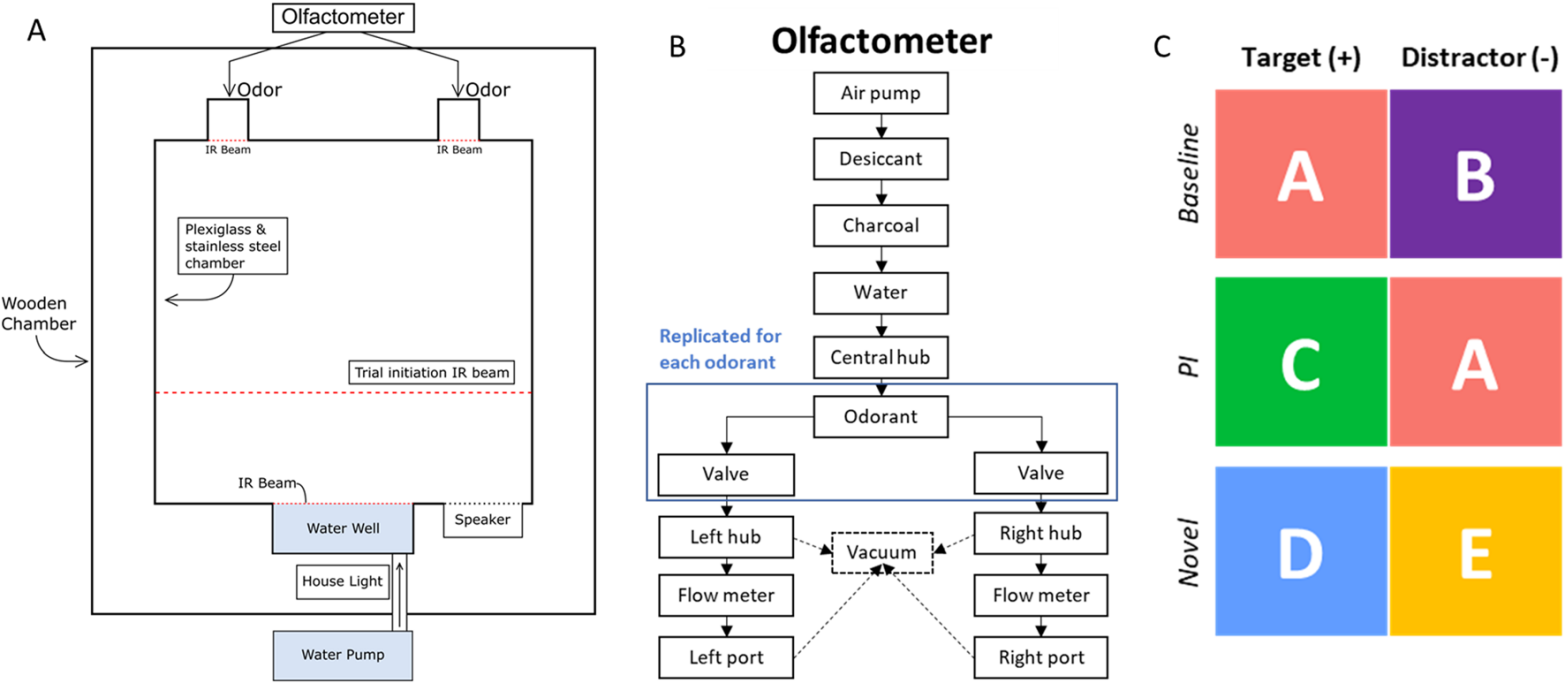
Apparatus and task design. **A** Operant chamber design. Note that odors could be readily detected from outside of the nosepoke aperture, so rats did not need to approach closely to sample the odors. **B** Example flow of olfactometer. After rehydration, air was distributed through a central hub to each of five odorant vials. When valves were open, odorized air rejoined the flow at a hub for each side, allowing control of odor and side of release. In between trials, air from each side hub and the ports was sent to vacuum. **C** Schematic of proactive interference task; colors and letters signify different odors. Targets (+) shown in left column and distractors (−) in right column, though in the task side was counterbalanced across trials

To investigate the effect of age on cognitive flexibility, we used an odor discrimination PI task in adult and aged rats and assessed the effect on cognitive flexibility of natural aging in conjunction with ACh manipulation via the nonspecific muscarinic ACh receptor antagonist scopolamine (SCOP), in contrast to the methylated molecule methylscoplamine (MSCOP) that is inefficient in crossing the blood brain barrier. In this task, adult and aged rats were rewarded for reporting the location of a target odor within an odor pair. We compared accuracy across two trial types: trials in which a new target stimulus was paired with a former target (now distractor) from a previously learned odor pair, thus eliciting PI; and trials of a novel pair in which neither odor in the pair had been experienced by the rats prior to testing (Fig. 1). This allowed us to independently assess encoding with interference and encoding without interference, respectively.

Previous studies across rats and human participants have demonstrated that this PI task depended on cholinergic action at central muscarinic receptors in the BF (Atri et al. 2004; Caplan et al. 2007; De Rosa et al. 2001, 2004; De Rosa and Hasselmo 2000). Conversely, encoding without interference did not rely on the cholinergic system. While these tasks have been presumed to model the contribution of ACh to PI resolution during natural aging, the present study directly tests this relationship by probing the intersectional effects of age and ACh manipulation on PI resolution and encoding novel associations.

This task is well-suited to detecting possible additive effects of age and cholinergic manipulation because the design allows detection of perseverative responses, i.e., subjects actively selecting the incorrect, but previously rewarded, odor in the interfering pair. Thus, chance-level does not represent a performance floor, providing a large range of possible effects on accuracy. Past work using this task found that increasing doses of SCOP in adult rats drove accuracy increasingly farther below chance for the interfering odor pair, reaching a minimum of approximately 30% accuracy (De Rosa and Hasselmo 2000). Indeed, perseveration has been commonly found in aging across humans and rodent models (Beas et al. 2017; Haaland et al. 1987; Ridderinkhof et al. 2002).

## Methods

### Animals

A total of 50 male Long-Evans rats (Charles River Laboratories, Wilmington MA) were used across two studies, consisting of 28 adult (mean age 9.8 months, S.D. 2.5 months) and 22 aged rats (mean age 21.8 months, S.D. 1.2 months). Of these, a minimum of 11 rats was used in each experimental group. Power analysis based on a previous study (De Rosa and Hasselmo 2000) using SCOP in adult rats suggested that a sample size of 9.6 animals, would allow us to detect even moderately sized drug effects with a power of 0.8, while a sample size of as few as 6.1 rats would allow us to detect differences between trial types at a power of 0.8.

Males were used to maximize comparability with past studies using this task, which also employed male rats exclusively (De Rosa et al. 2001, 2004; De Rosa and Hasselmo 2000). All rats were housed, and all experiments conducted, in accordance with the Cornell University IACUC. Rats were kept on a 12-h reverse day/night schedule and placed on water restriction for the duration of training and testing, wherein rats received 15 min of free water following training or testing each day. Weighing three times weekly ensured no rat fell below a minimum of 80% of individual starting weight during water restriction, with no maximum weight imposed. Prior to start of shaping, rats acclimatized to the vivarium for seven days and then were handled daily for an additional 5–10 days until fully habituated to the researchers.

### Behavioral apparatus

All training and testing occurred within a 25 cm x 25 cm x 25 cm plexiglass and stainless-steel operant chamber (Med Associates Inc., Fairfax VT) inside a ventilated noise-reducing wooden chamber (Fig. 1A). One inner face of the operant chamber was equipped with 2.5 cm square apertures that served both to release odor, through a custom-designed olfactometer connected outside the chamber, and to record rats’ nose poke responses through breaking an infrared beam at the front of the aperture. The opposing face of the chamber held a water well with an infrared beam at the entry and an adjacent reward indication light, with a house light and speaker near the top of the chamber. An additional infrared beam outside the water well was used to initiate trials.

A custom olfactometer (Fig. 1B) delivered the odorized air to the operant chamber at 1.5 L per minute. Air entered via a pump and was dehydrated, filtered, and rehydrated before passing to vials containing individual odorants. Odorized air was directed to the operant chamber as appropriate in the task paradigm via 3-way solenoid valves. Between trials air from the olfactometer and from the operant chamber apertures was evacuated by a vacuum pump. All trial events were controlled and recorded by a custom Python program via an input/output interface card (Med Associates Inc., Fairfax VT).

### Proactive interference task and training

We elicited proactive interference using a paired associate odor discrimination task employed previously (De Rosa & Hasselmo 2000; De Rosa et al. 2001). In this task, rats were presented simultaneously with two odors that comprised an odor pair: a target and a distractor. The rats were required to learn which odor in each pair was the target through trial and error, indicating responses by poking the port releasing the target odor. In our operant chamber odors are easily detected from some distance, so rats did not need to engage the nosepoke to sample which odor was on which side but rather could determine target odor location as they approached. The stimulus–response associations of the odors could then be experimentally manipulated to challenge learning, e.g., by inducing PI.

Rats first underwent shaping in which they were taught to initiate trials by breaking an infrared beam at the rear of the operant chamber, then taught to nosepoke either of the ports at the opposite end of the chamber to obtain a water reward. A green LED light above the water well indicated reward availability; breaking the infrared beam at the water well entrance triggered reward delivery. Once rats learned to perform the nosepoke response they were taught to discriminate first between odorized air vs. non-odorized air, then between pairs of odors. At each stage rats were first allowed to make as many attempts as needed to select the correct port; subsequently as rats mastered each stage negative feedback was added such that incorrect responses simultaneously triggered a mildly aversive error tone and the house light. Rats were advanced to the next shaping sage when they could achieve a behavioral criterion (90% correct in any 20-trial period or > 75% overall for at least two daily sessions) in session of 64 trials, which typically required approximately seven to 10 days at each stage.

Odor stimuli were plant-based essential oils and fragrances diluted in mineral oil (Bulk Apothecary, Aurora, OH), selected to be clearly distinguishable as determined by a human experimenter while neither inherently aversive nor appetitive (for example, no odors that mimicked scents of predators or food sources were used, rather scents were mostly floral or fruit scents; Table 1). Odor sets were counterbalanced across drug conditions to reduce any influence of the individual stimuli on the results. Unless otherwise stated, odorants were applied at full concentration; particularly potent odorants were mixed in pure mineral oil (Sigma Aldrich, St. Louis, MO).

**Table 1:** Odor stimuli. All of the stimuli were plant-based oils

	Odor set 1	Odor set 2	Odor set 3
Baseline target (A)	Tomato flower fragrance	Vanilla fragrance	Ginger root essence
Baseline distractor (B)	Banana fragrance	Peppermint essence, diluted 2:1 with mineral oil	Spearmint essence
PI target (C)	Cinnamon bark essence	Caraway essence	Coffee essence
Novel target (D)	Carnation fragrance	Marjoram essence	Cuban tabacco fragrance
Novel distractor (E)	Mango Madness fragrance	Lemongrass essence, diluted 1:1 with mineral oil	Palmarosa essence

Each odor set was selected such that all five odors within an odor set were discriminable and odors were distinct between sets. A new odor set from among those listed was used for each drug condition, i.e., an individual rat did not repeat odor sets or odor associations, and the drug conditions were counterbalanced across odor sets

Immediately prior to experimental testing, rats were taught a baseline odor pair (e.g., A + / B–, where + indicates a rewarded target stimulus and—indicates a non-rewarded distractor stimulus) over a series of sessions until proficient in indicating the target of this baseline pair, as measured by meeting the aforementioned behavioral criteria.

Experimental testing consisted of one session per day for five consecutive days. Each session began with 32 “reminder” trials of the previously learned baseline pair, then proceeded to interleaved trials of two types: 32 proactive interference (PI) trials in which the target from the baseline pair became the distractor (C + /A−), and a new stimulus is the target (encoding with interference); and 32 Novel trials consisting of two stimuli without prior association (D + /E−) (encoding without interference) for a total of 96 trials a day (Fig. 1C). To succeed in PI trials rats must overcome past learning about a former target stimulus; thus, proactive interference is elicited. The Novel trials allowed us to contrast PI resolution with encoding in the absence of interference.

Performance was measured in accuracy for each session, i.e., the percentage of trials of each type in which the rat selected the correct odor, without counting omissions (trials where the rat made no response in the 30-s window). Accuracy was measured separately for each trial type within each session. This generous time window allowed rats to respond accurately with no need to make speeded responses; as such, reaction time is not a sensitive measure in this task.

### Drugs and injection

Rats received intraperitoneal injections of normal sterile saline during initial learning of the baseline pairs to acclimate them to injection. During testing, the nonspecific muscarinic antagonist scopolamine (Krackler Scientific, Albany, NY) or its methylated counterpart, methylscopolamine (Krackler Scientific, Albany, NY), dissolved in 9% sterile saline (Krackler Scientific, Albany, NY), were administer via I.P. injection at 0.25 m.g./ k.g. body weight 20 min prior to behavioral testing. Methylscopolamine has limited ability to cross the blood brain barrier and served as a control substance allows isolation of the central effects of ACh at the muscarinic receptors. We selected this dosage and administration paradigm based on previous work demonstrating the selective impaired of interference resolution under these conditions, whereas lower doses do not effect interference resolution and higher doses non-selectively impact new learning as well as learning with interference (De Rosa and Hasselmo 2000). To allow within-subjects comparisons of ACh blockade, each rat underwent testing in one drug condition, then learned a new baseline odor pair and completed a second round of testing, with new odor sets, in the other drug condition. Drug order was counterbalanced across rats, and odor sets, and the experimenter was blind to drug condition.

### Statistical analysis

All statistical analyses were performed in R (R Core Team 2019). Accuracy was assessed via mixed effects models (Bates et al. 2015) with accuracy as the dependent measure, rat identity as a random effect and session (1–5), drug (MSCOP or SCOP), trial type (Baseline, PI, or Novel), and age (adult or aged) as fixed effects. Output of the mixed models was submitted to a type III sums of squares F test with Kenward Roger estimation of degrees freedom (Kenward et al. 1997). Mixed models typically employ estimates of degrees freedom that attempt to account for the impact of covariance within the data (Bolker 2020), rather than traditional deterministic measured of degrees freedom. Our analysis utilized the *lme4* (Bates et al. 2015) and *car* (Gajewski et al. 2010) packages in R. Past work has demonstrated the applicability of this approach for generalized linear mixed models as were used in this study (Luke 2017; Stroup 2013, 2015). Post-hoc analysis used pairwise t-tests with a Holm correction for repeated testing.

## Results

We first performed a pilot experiment to confirm that age alone influenced cognitive flexibility in our task, comparing PI resolution in adult (*n* = 17, mean age 9.8 months, S.D. 3.1 months) and aged (*n* = 11, mean age 23.0 months, S.D. 0.0 months) rats with no pharmacological manipulation. The adult group comprised two cohorts of rats tested at separate times; because direct comparison indicated no difference in performance between these two groups (*F*(1,14.907) = 0.178, *p* = 0.679), we proceeded with these cohorts pooled for further analyses.

We used a mixed effects model with age, trial type, and day as fixed effects. As expected, both age groups exhibited behavior consistent with proactive interference (PI), demonstrated in a main effect of trial type across all five testing sessions and both age groups (*F*(1,231.090) = 60.412 *p* < 0.001) that was driven by significantly lower accuracy in the PI trials compared to Novel trials (mean_PI_ = 66.9%, mean_Novel_ = 78.7%). Thus, the interference manipulation was effective in both ages.

To determine whether the aged and adult rats differed in their eventual resolution of PI, we compared accuracy in PI trials vs. Novel trials specifically in the final testing session. At this late stage, adult rats no longer showed a significant difference in accuracy between these odor pairs (*t*(25) = 1.321, *p* = 0.195), indicating they had overcome interference. Conversely, aged rats continued to perform significantly worse in PI trials compared to Novel (*t*(25) = 4.33, *p* < 0.001), revealing that aged rats were impaired in efficient PI resolution. This indicated that our task is sensitive to declines in cognitive flexibility commonly associated with age (Barense et al. 2002; Harada et al. 2013; Head et al. 2009; Lacreuse et al. 2018), laying the foundation for our subsequent examination of the interactions between age and muscarinic receptor antagonism.

### Natural aging and muscarinic receptor blockade

Our pilot experiment demonstrated that natural aging impaired the resolution of PI, extending past evidence suggesting that ACh is involved in this form of cognitive flexibility (Atri et al. 2004; De Rosa et al. 2001; De Rosa and Hasselmo 2000). Next, we assessed the relationship between function of the cholinergic system at the muscarinic receptors and the observed age effects. To test this, we repeated the odor discrimination PI task in new cohorts of adult rats (*n* = 12, 9.8 months, S.D. 1.3 months) and aged rats (*n* = 11, mean age 20.7 months, S.D. 0.5 months). We found that systemic SCOP and MSCOP at the doses used previously (De Rosa and Hasselmo 2000) was poorly tolerated in extremely aged rats, resulting in extreme lethargy, immobility, and disengagement from the behavioral task, so for these experiments we used an aged group that was two months younger than those in our pilot study.

We investigated whether natural aging altered the impact of cholinergic antagonists on rats’ ability to overcome interference by modeling adult and aged rats together and probing the effects of age, trail type and drug on accuracy. There was no main effect of age (*F*(1,21) = 1.275, *p* = 0.272), indicating that aging did not globally impair learning. Rather, the effect of trial type, i.e., the reduced accuracy in PI trials compared to Novel, was significantly larger in the aged rats compared to the adults (*t*(375.000) = 12.505, *p* < 0.0001), indicating their greater susceptibility to interference without a global deficit in learning. A main effect of trail type demonstrated the overall impact of conflict from past learning in both age groups (*F*(1, 375) = 156.371, *p* < 0.0001, mean_PI_ = 51.6%, mean_Novel_ = 76.4%). In comparison, the main effect of drug when pooled across trial types and ages, while significant (*F*(1, 376) = 8.426, *p* = 0.004), was small mean_MSCOP_ = 66.2%, mean_SCOP_ = 61.7%, which was unsurprising given past evidence that SCOP only strongly impacts interference resolution but not new learning.

Replicating past findings, SCOP significantly reduced PI trial accuracy in adult rats (*t*(375.000) = 2.940, *p* < 0.01); however, there was no such drug effect on PI accuracy for aged rats (*t*(376.000) = 1.200, *p* = 0.231). To directly assess the relative impact of SCOP across ages, we performed a focused contrast comparing the effect of drug on PI accuracy in adult vs. in aged rats. This revealed that the drug effect was significantly reduced in the aged cohort compared to the adult group (*t*(376.000) = 2.881, *p* < 0.01).

SCOP did not impact accuracy on Novel trials in either adult (*t*(375.000) = 0.313, *p* = 0.755) or aged rats (*t*(376.000) = 1.391, *p* = 0.165), nor was there a significant difference between age groups in the SCOP effect on Novel accuracy, (*t*(376.000) = 1.235, *p* = 0.218), demonstrating that cholinergic antagonism did not disrupt learning free from interference regardless of age.

**Fig. 2:**
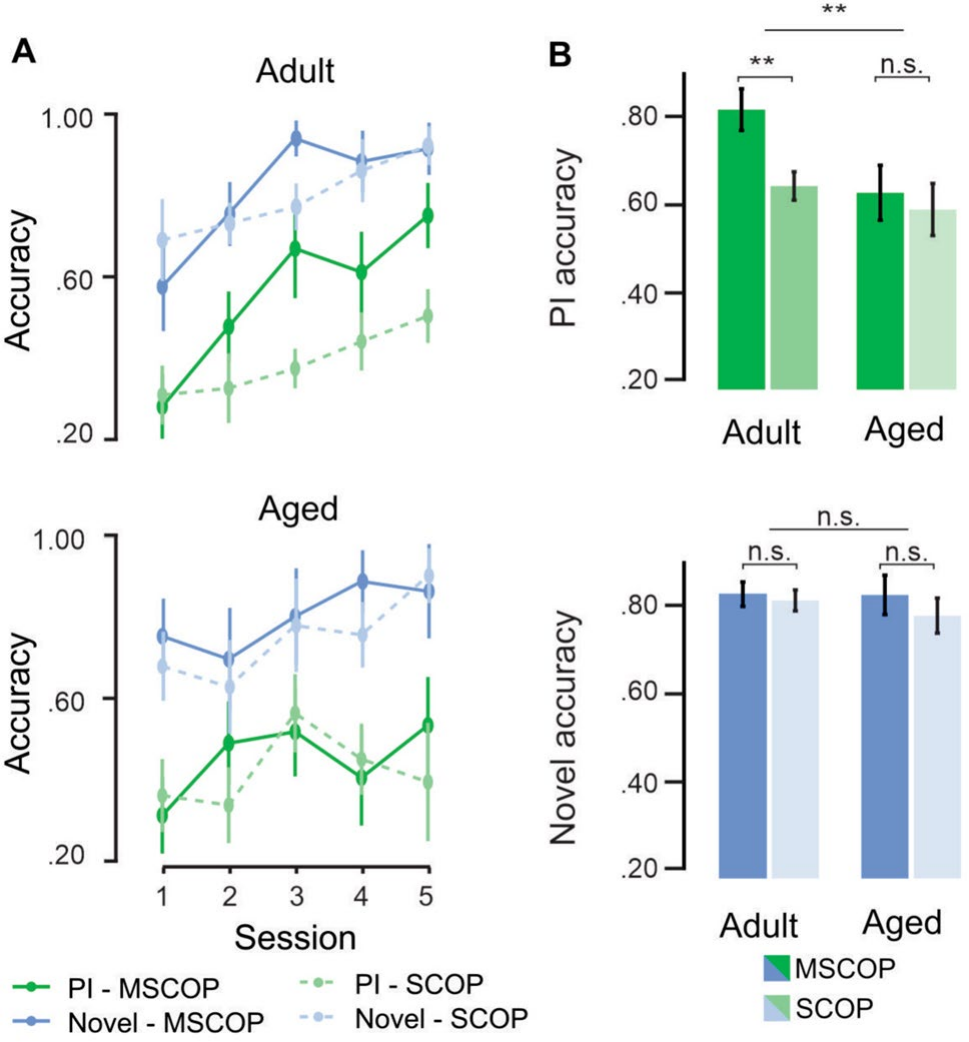
The effects of drug condition and age on novel learning and interference resolution. A) Learning trajectories of adult (top) and aged (bottom) rats for Novel (blue) and PI (green) trials under MSCOP (solid lines) and SCOP (dashed lines). Under the MSCOP control, adult mice gradually increase accuracy for both PI and Novel trials, with no difference in accuracy between trial types by the final testing day; under SCOP PI accuracy remains depressed compared to Novel accuracy throughout testing. Aged mice do not show eventual PI resolution under either drug condition. B). Drug effect on PI accuracy (top) and Novel accuracy (bottom) for adult and aged rats, averaging over all testing days. Adult rats, but not aged rats, have significantly lower PI trial accuracy under SCOP; direct contrast (uppermost significance bar) shows that SCOP has a significantly greater effect in adult vs. aged rats. Neither age group showed a drug effect for Novel trial accuracy, nor was the difference in drug effect across age groups significant. Error bars indicate S.E.M. **p < 0.01; N.S. = not significant

We further probed eventual resolution of interference by comparing accuracy in PI trials vs. Novel trials specifically in the final testing session. Adult rats under MSCOP had resolved interference at this late stage, showing no significant difference in accuracy between these two trial types (*t*(58.3) = 1.376, *p* = 0.174, Fig. 2A, top), while under SCOP a significant difference in accuracy persisted types (*t*(58.3) = 3.508, *p* < 0.001). Conversely aged rats had significantly lower accuracy in PI trials compared to Novel at the final test session both under MSCOP (*t*(58.3) = 3.187, *p* < 0.01, Fig. 2A, bottom) and under SCOP (*t*(62.1) = 4.169, *p* < 0.0001).

These difference in PI learning and in sensitivity to SCOP were not attributable to differences in weight loss due to water deprivation, which may conceivably have acted on motivational state, as the age groups maintained similar fractions of initial body weight over the course of testing – indeed both age cohorts gained small fraction of initial weight, on average (*t*(20.5) = 0.937, *p* = 0.360, mean_Adult_ = 106% of initial weight maintained at end of testing, mean_Aged_ = 110%).

Thus, the present data replicated past results in showing that in adults rats, SCOP impairs the ability to resolve interference while sparing novel learning. Moreover, these data demonstrate that, while natural aging under pharmacological control conditions reduced rats’ ability to encode new information when experiencing interference, aged rats were considerably less susceptible than adult rats to cholinergic antagonism.

## Discussion

In the present study we examined how the cholinergic neuromodulatory system contributes to cognitive flexibility over the lifespan, probing the interacting effects of natural age and central muscarinic receptor blockade on rats’ ability to retrieve past learning and to encode new information with or without interference from retrieval. While previous research has examined the independent influences of natural aging and cholinergic disruption, we combined these manipulations to probe whether ACh is employed similarly during PI resolution across adult and aged rats.

We postulated that decreasing function of the BF cholinergic system at central muscarinic receptors is at least partly responsible for age-related challenges to cognitive flexibility (Barense et al. 2002; Bizon et al. 2012). Consistent with this, we found that aged rats, in comparison to adult rats, were inefficient at resolving interference from previously learned associations when acquiring new, overlapping information.

In adult rats, impairing ACh action at central muscarinic receptors with SCOP selectively reduced interference resolution, but not novel learning (De Rosa and Hasselmo 2000). Surprisingly, in aged rats there was no effect of muscarinic antagonism. Importantly, aged rats’ performance near chance in the interfering odor pair under the MSCOP leaves considerable room for further impairment in performance: animals demonstrating perseveration can perform well below chance in this task as they persistently choose the incorrect odor that previously served as a target (De Rosa and Hasselmo 2000). Thus, our findings indicate that cholinergic antagonism differentially impacted aged and adult rats, specifically in their capacity to resolve interference.

There is conflicting past research on the magnitude of SCOP effects in aged compared to adult subjects (Appenroth and Fleck 2010; Flicker et al. 1992; Spangler et al. 1989; Tariot et al. 1996). Intriguingly, aging and lesion studies demonstrating increased sensitivity to SCOP typically do not see any performance deficit from age or lesion alone (Sarter and Bruno 1998), whereas studies that report no increased sensitivity to SCOP conversely do see robust effects of age separate from pharmacology manipulations. Gage and Bjorklund (1986) found that only aged rats with no memory impairment relative to adult animals were sensitive to SCOP, whereas aged rats whose performance was worse than adult rats were insensitive to SCOP. Similarly, both age (Mandairon et al. 2011) and basal forebrain lesion (Aigner et al. 1987) reduce behavioral sensitivity to the acetylcholinesterase inhibitor physostigmine, despite the drug increasing ACh activity. These results suggest that external manipulation of ACh may be less impactful when natural cholinergic loss has already functionally weakened behavior.

While our current data cannot directly elucidate the mechanisms underlying the observed results, speculatively one explanation of our results and this past literature is that aged animals represent a low-ACh state, where the reduced cholinergic modulation is already captured in age-related reductions of performance, and any remaining performance is due to compensation that uses alternative mechanisms besides ACh. For example, older adult humans rely more on frontal cortices to parse relevant from irrelevant stimulus information, compensating for a reduced capacity for sensory cortices to perform such filtering, potentially due to cholinergic loss (Schmitz et al. 2014). In such a scenario, SCOP would have little effect because performance no longer relies on ACh.

A limitation of the present experiment is that our use of only male rats, while consistent with past research (De Rosa et al. 2001; De Rosa and Hasselmo 2000), prevented us from assessing sex differences in interference resolution over the life course. Several previous studies indicate that male rats show greater age-associated cognitive decline than female rats, and that this may correspond with more pronounced degradation of the BF cholinergic system (Lukoyanov et al. 1999; Veng et al. 2003; Zhvania et al. 2021). Conversely, large scale studies of human subjects find that females express more rapid cognitive decline than males, highlighting the complex relationship between sex and cognitive aging and the need for continued focus in this area (Levine et al. 2021; Lipnicki et al. 2017). Future work should probe how sex interacts with age and the BF system in interference resolution.

We have demonstrated that age profoundly challenges rats’ cognitive flexibility in integrating new learning with past associations, and that aged rats differ profoundly from adult rats in their sensitivity to cholinergic disruption in this process. Further research is needed to clarify how changes in ACh transmission in the cortices alter the neural systems supporting cognitive flexibility over the lifespan.

## Data Availability

The data that support the findings of this study are available from the corresponding authors upon request.

## Abbreviations

BF: Basal forebrain
PI: Proactive interference
SCOP: Scopolamine
MSCOP: Methylscopolamine
